# Pharmacologically relevant doses of valproate upregulate CD20 expression in three diffuse large B-cell lymphoma patients in vivo

**DOI:** 10.1186/2162-3619-4-4

**Published:** 2015-01-26

**Authors:** Jesper Kofoed Damm, Sandra Gordon, Mats Ehinger, Mats Jerkeman, Urban Gullberg, Anne Hultquist, Kristina Drott

**Affiliations:** Department of Hematology and Transfusion Medicine, Lund University, Lund, Sweden; Department of Pathology, Skåne University Hospital, Lund, Sweden; Department of Oncology, Skåne University Hospital, Lund, Sweden; Stem Cell Center (SCC), Lund University, Lund, Sweden

**Keywords:** Valproate, Valproic acid, CD20, DLBCL, Rituximab, HDACi

## Abstract

**Background:**

Epigenetic code modifications by histone deacetylase inhibitors (HDACi) have been proposed as potential new therapies for lymphoid malignancies. Diffuse large B-cell lymphoma (DLBCL) is the most common type of aggressive lymphoma for which standard first line treatment is the chemotherapy regimen CHOP (cyclophosphamide, doxorubicin, vincristine and prednisone) combined with the monoclonal anti-CD20 antibody rituximab (R-CHOP). The HDACi valproate, which has for long been utilized in anti-convulsive therapy, has been shown to sensitize to chemotherapy *in vitro*. Valproate upregulates expression of CD20 in lymphoma cell lines; therefore, 48 hour pre-treatment with valproate before first line R-CHOP in DLBCL stages II-IV is evaluated in the phase I clinical trial VALFRID; Valproate as First line therapy in combination with Rituximab and CHOP in Diffuse large B-cell lymphoma.

**Findings:**

Pretreatment with valproate at oral doses comparable to anti-convulsive therapy, resulted in upregulation of CD20 mRNA and CD20 protein on the cell surface as measured by qPCR and FACS analysis in lymphoma biopsies from three evaluated patients from the VALFRID study. Valproate-treatment corresponded to increased acetylation of Histone3Lysine9 (H3K9ac) in peripheral blood mononuclear cells (PBMCs), which were employed as surrogate tissue for valproate-related epigenetic modifications.

**Conclusions:**

Valproate treatment at pharmacologically relevant doses resulted in upregulation of CD20 *in vivo*, and also in expected epigenetic modifications. This suggests that pre-treatment with valproate or other HDACis before anti-CD20 therapy could be advantageous in CD20-low B-cell lymphomas. Further studies are warranted to evaluate this conclusion.

## Introduction

Diffuse Large B-cell Lymphoma (DLBCL) is an aggressive B-cell lymphoma. Recent results show that inactivating mutations of the histone acetyltransferases EP300 and CREBBP*,* and of the histone methyltransferases MLL2 and EZH2 occur in the majority of DLBCL cases [1–3]*.* Hence, new treatments in DLBCL should aim at restoring physiologic acetylation and methylation levels and the use of epigenetic therapy could therefore have a rational basis in DLBCL. At present, standard first line treatment of DLBCL is chemotherapy consisting of a combination of cyclophosphamide, doxorubicin, vincristine and prednisone (CHOP). During recent years addition of the anti-CD20 antibody rituximab has become an international clinical standard (R-CHOP) leading to an improved progression-free, event-free, disease-free and overall survival. Although R-CHOP leads to remission in 85% of patients, about 50% of these relapse, often with disease that is resistant to rituximab [4].

A possible mechanism for resistance to antibodies targeting CD20 is transcriptional downregulation of CD20 mRNA through epigenetic mechanisms [5, 6]. Indeed, Shimizu et al. have shown that histone deacetylase inhibitors such as valproate and romidepsin can increase acetylation of the CD20 promoter resulting in recruitment of the Sp1 transcription factor and increased expression of CD20 mRNA and protein in B-cell lymphoma cell lines [7]. However, to our knowledge these findings have so far not been extended to clinical trials.

## Results

### Valproate upregulates CD20 expression in three diffuse large B-cell lymphoma patients

**Figure 1:**
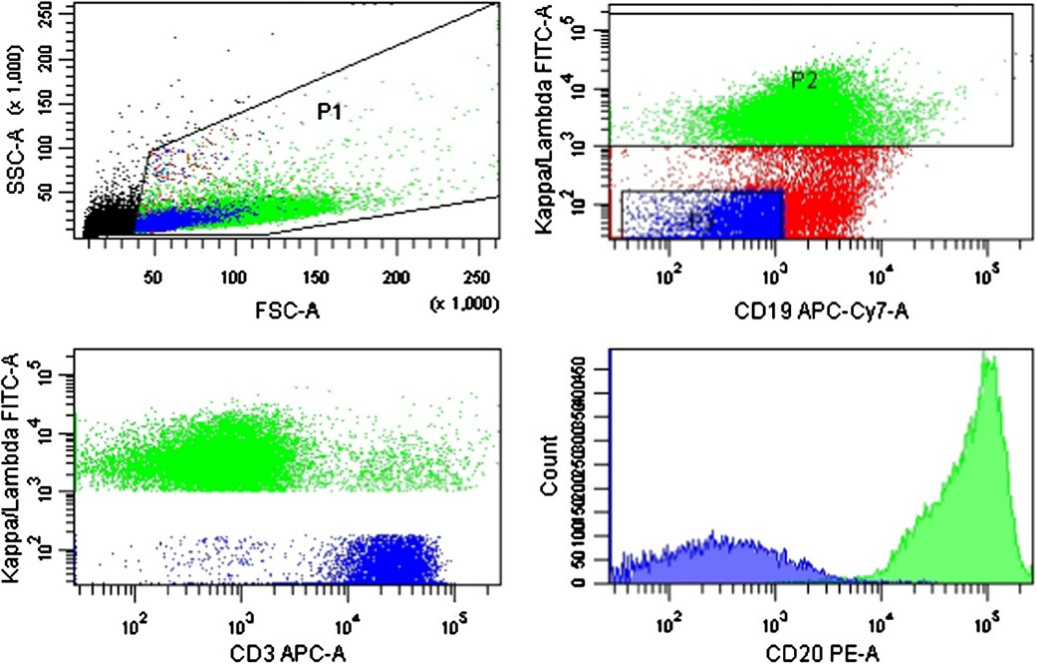
**FACS gating and CD20 expression analysis of lymphoma cells.** Lymphoma cells were defined by either kappa or lambda monoclonal CD19+/CD3- cells. Histograms of detected CD20 were used for quantification of bound anti-CD20 mAbs per cell by QuantiBRITE assay as presented in Table 2. The figure shows the analysis of patient 008, day 0 as a representative example.

In 2001 *valproate*, a gamma-aminobutyric acid (GABA) agonist with a long history of clinical use for treatment of epilepsy and mood disorders (reviewed in [8]), was identified having HDAC inhibitory activity. We have previously developed an experimental model in which 48 hour pretreatment with valproate at pharmacological doses strikingly sensitises diffuse large B-cell lymphoma cell lines to R-CHOP induced apoptosis. Moreover, in this model the combination of valproate and prednisolone has a synergistic effect on R-CHOP-induced cell death [9]. Based on these findings, we have initiated a phase I trial with a dose expansion at the recommended phase II dose (VALFRID: Valproate as First line therapy in combination with Rituximab and CHOP in Diffuse large B-cell lymphoma [10]). VALFRID is currently on-going at three University Hospitals in Sweden, and aims at including 35 patients. In this trial valproate is administered orally three times daily prior to R-CHOP days 1–3 as first line treatment of DLBCL patients stages II-IV. In the VALFRID trial, prednisone from the R-CHOP regimen (75–100 mg daily) is administered day 1–5 (together with valproate day 1–3) and rituximab, cyclophosphamide and doxorubicin on day 3 (Table 1)*.* In the dose-escalation part of the study, three consenting patients (i.e., patients 003, 008 and 010) underwent a fine needle biopsy (FNB) from an affected lymph node before start of valproate/prednisone on cycle 1 day 0, and a repeated biopsy after 48-hour treatment the morning on day 3 (i.e., before start of R-CHO). In this material, upregulation of CD20 protein on the cell surface of lymphoma cells was assessed by flow cytometry analysis and upregulation of CD20 mRNA by qPCR. A representative example of the utilised flow cytometry gating for the sorting is shown in Figure 1.

**Table 1:** **Overview of drug and sampling administration in the VALFRID study**

Day	0	1	2	3	4	5
Valproate		+++	+++	+++		
Prednisone		+	+	+	+	+
R-CHO				+		
PBMC	+			+		
FNB	+			+		

Valproate was administered three times daily day 1–3, prednisone was administered day 1–5 and R-CHO (rituximab, cyclophosphamide, doxorubicin and vincristine) was administered day 3. PBMCs were collected in the morning day 0 and day 3 of the 1st, 3rd and 6th treatment cycles before the R-CHO treatment was administered. Fine needle biopsy was also performed on day 0 and in the morning of day 3 before R-CHO treatment was administered in the 1st treatment cycle of consenting applicable patients.

All patients were treated with pharmacologically relevant doses of valproate comparable to those utilised in antiepileptic treatment. For antiepileptic therapeutic purposes plasma levels of 300 to 700 μM is desired [11]. In the VALFRID study, valproate treatment resulted in serum levels between 400–850 μM (please see Table 2 for doses and serum levels of valproate in patients undergoing an FNB). The number of bound anti-CD20 molecules per cell surface was measured by standardizing geometric mean fluorescence intensities (MFI) through results from a QuantiBRITE® assay (beads covered with known quantities of bound PE).

**Table 2:** **Number of bound CD20 antibodies per lymphoma cell before and after valproate treatment**

***Patient***	***Valproate dosage*** (mg/kg/day)	***Day 0***		***Day 3***		
		***MFI***	***Bound CD20 mAbs per cell***	***MFI***	***Bound CD20 mAbs per cell***	***Plasma valproate (μM)***
**003**	30	2163	6866	7602	18035	*407
**008**	80	30893	70229	34322	78890	656
**010**	80	35540	69096	41605	79260	847

An FNB of an affected lymph node was performed before treatment start as well as morning day 3, cycle 1 in patients 003, 008 and 010 of the VALFRID study. Lymphoma cells (i.e., monoclonal B-cells) were analysed by FACS as described in materials and methods, and the number of bound CD20 antibodies to the cell surface was calculated by normalisation by the QuantiBRITE assay using geometric mean fluorescence intensity (MFI). Corresponding doses and serum levels of valproate are indicated (*: Day 2).

**Figure 2:**
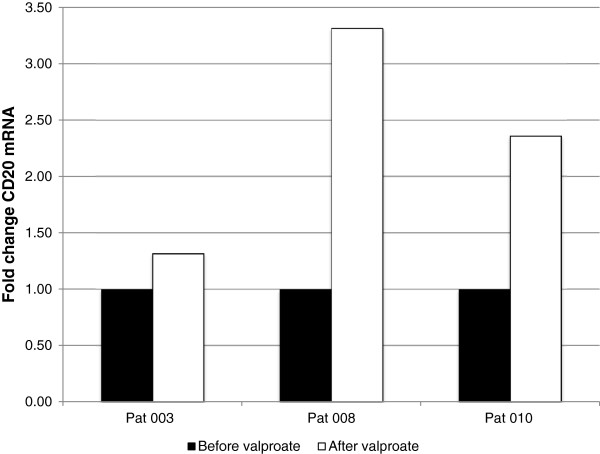
**Fold change of CD20 mRNA in lymphoma cells after valproate treatment.** A fine needle biopsy of an affected lymph node was performed before treatment start as well as morning day 3, cycle 1 in patients 003, 008 and 010 of the VALFRID study. The lymphoma cells (i.e., monoclonal B-cells) were sorted by FACS as described in materials and methods. Levels of CD20 mRNA were estimated by qPCR. For dosage and serum-levels of valproate, please see Table 2.

Already at a valproate dose of 30 mg/kg/day (given to patient 003) a slight increase of CD20 mRNA was measured on day 3 (Figure 2). This was correlated to a three-fold increase in CD20 molecules exposed on the cell surface (Table 2)*.* Valproate at 80 mg/kg/day resulted in a more robust increase in levels of CD20 mRNA. However, the increase of CD20 molecules on the cell surface was more modest, possibly explained by the high base line expression of CD20 on the cell surface of these patients.

**Figure 3:**
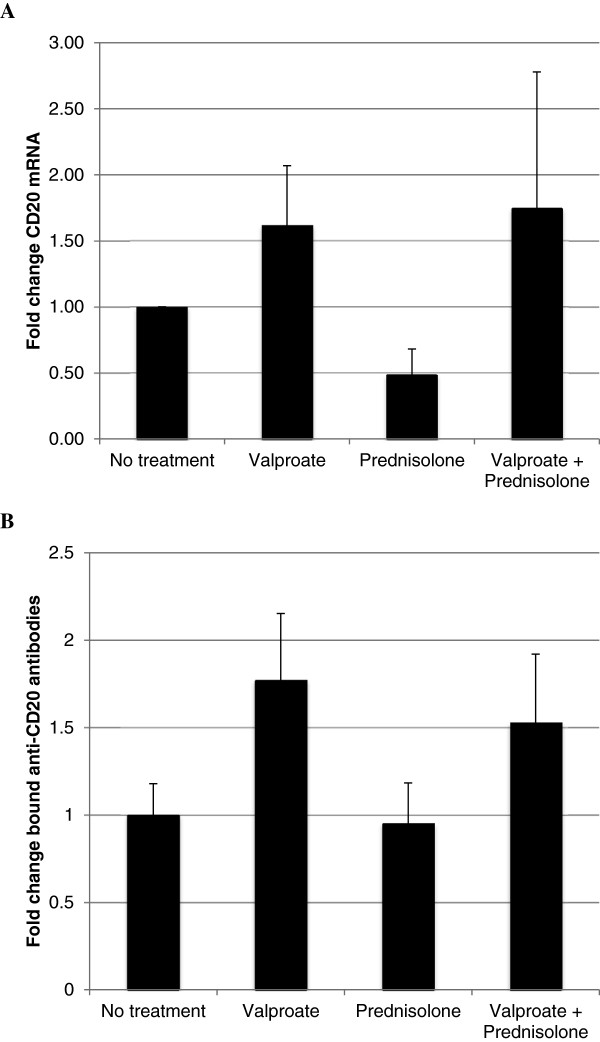
**Effects of combination therapy with valproate and prednisolone in SU-DHL-8 cells.** SU-DHL-8 cells were incubated with or without 1 mM valproate and/or 55 μM prednisolone in cell culture media. After 48 hours, cells were harvested and levels of CD20 mRNA were estimated by qPCR **(A)**. Quantification of anti-CD20 antibodies bound to the cell surface was estimated using FACS and QuantiBRITE assay **(B)**. Mean values are from five separate experiments, bars represent standard deviation.

Since prednisone was administered together with valproate, possible prednisone-related effects on CD20 expression were evaluated in the DLBCL cell line SU-DHL-8. However, as shown in Figure 3, while incubation with 1 mM of valproate resulted in prompt induction of CD20 in these cells, no prednisone-related effects on either CD20 mRNA or cell surface protein were observed. This speaks against prednisone-related effects on CD20 expression, and supports that valproate significantly upregulates CD20 expression both on the mRNA level and on the cell surface in diffuse large B-cell lymphoma patients**.**

### Valproate-related effects in surrogate tissue

**Figure 4:**
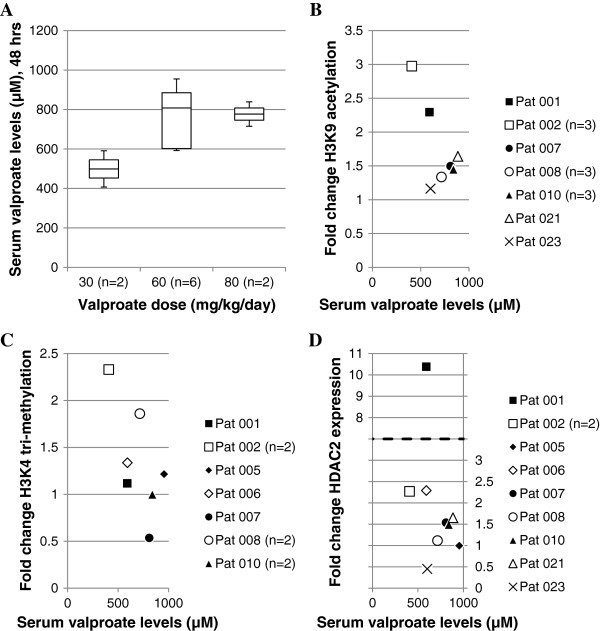
**Serum valproate levels and fold change in epigenetic biomarkers of surrogate tissue. (A)** Serum valproate levels in response to 48-hour treatment of valproate. **(B-D)** Fold change of H3K9ac, H3K4me3 and HDAC2 expression in PBMCs as judged by Western blot. Blots were quantified by normalising the epigenetic biomarker to the corresponding GAPDH expression and fold change determined by normalisation to Day 0 sample. Samples are from cycle 1, 3 and 6.

To assess whether the utilised doses of valproate resulted in expected histone modifications, peripheral blood mononuclear cells (PBMCs) were employed as a model. Acetylation of lysine 9 of histone H3 (H3K9ac) in PBMCs has been suggested as an adequate surrogate tissue marker for the HDAC inhibitory activity of valproate in tumour cells *in vivo*[12]. Moreover, 5 mM valproate has been shown to affect the tri-methylation of lysine 4 of histone H3 (H3K4me3) *in vitro*[13]. Therefore, H3K9ac and H3K4me3 were studied by Western blot in available PBMCs from patients 001, 002, 005, 006, 007, 008, 010, 021, 023 from the VALFRID study. These patients were treated with valproate between 30–80 mg/kg/day, resulting in serum levels between 400–1,000 μM (Figure 4A)*.* As shown in Figure 4B, valproate treatment resulted in an increase in levels of H3K9ac already at serum levels of 400 μM, suggesting that levels of valproate were sufficient to achieve expected histone acetylation. H3K4me3 was increased in five patients, unchanged in one and reduced in two patients (Figure 4C).

Although valproate has been suggested as a general inhibitor of class I and class IIa HDACs [8], isolated knockdown of HDAC2 mimics valproate-related effects in preclinical models, and levels of HDAC2 have been suggested as a relevant therapeutic target during valproate treatment *in vivo*[12, 14, 15]. However it is not known whether a therapeutic response to valproate treatment correlates to up- or downregulation of HDAC2. Therefore, levels of HDAC2 during valproate treatment of PBMCs were monitored by Western blot but no certain conclusions could be made to the HDAC2 levels in response to valproate in this study (Figure 4D).

## Discussion

Although the sample size is small, our data suggest that pharmacologically relevant doses of valproate may upregulate CD20 expression in DLBCL patients. To our knowledge this has not been shown in an *in vivo* situation before. Given that CD20 expression levels may be a limiting factor for rituximab response, the results support that valproate pretreatment could be beneficial for the response to anti-CD20 treatment, particularly in cases with low expression of CD20. Not only could relapsing DLBCL with low levels of CD20 benefit from valproate treatment prior to therapy targeting CD20, but it is also possible that valproate treatment could enhance the efficacy of anti-CD20 antibodies in chronic lymphocytic leukaemia, a B-cell lymphoma expressing low to intermediate levels of CD20 [16].

The doses of valproate utilised are in the range of what is prescribed during continuous anti-epileptic treatment and still, effects are observed on PBMC H3K9ac as measured by Western blot. The effect on PBMC H3K4me3 is less evident, but may still suggest clinically relevant valproate-related effects on histone trimethylation. However, the effect of valproate on histone-methylation is probably weaker, as compared to that on histone-acetylation, consistent with the previous notion that valproate-levels 5–10 times higher than the actual serum levels were shown to induce histone tri-methylation *in vitro*[13].

The valproate administered was given together with prednisone of the R-CHOP regimen. It cannot be excluded that this may skew the obtained results. However, we do not observe a prednisolone-induced upregulation of CD20 *in vitro*, suggesting that the observed upregulation of CD20 is indeed related to valproate.

Taken together, our data suggest that administration of valproate before anti-CD20 treatment may result in increased response to anti-CD20 treatment, and also may be feasible and safe. Indeed, this notion will be further explored in PREVAIL, a phase 0 trial on CLL patients starting the fall of 2014 at Skåne University Hospital [17].

## Materials and methods

### Eligibility

Please see ClinicalTrials.gov: ID NCT01622439 for eligibility for the VALFRID study. Informed consent was obtained from patients in accordance with good clinical practice and federal and institutional guidelines governing registered clinical trials [10]. The fine-needle biopsy of an affected lymph node before and after valproate was optional, and patients 003, 008 and 010 all signed an additional consent form for research on material from the fine-needle biopsy.

### Study treatment

Valproate (Ergenyl® or Orfiril®) was administered orally every 8 hours for 3 days at doses indicated above. Prednisone was administered day 1–5. R-CHO (rituximab, cyclophosphamide, doxorubicin and vincristine) was given after the morning dose of valproate on day 3 according to standard protocol.

### Pharmacokinetics

Levels of total plasma valproate were measured before morning dose of valproate day 2, 3 and 4 by a homogenous enzyme-linked immunoanalysis technique at the Department of Clinical Chemistry at Skåne University Hospital.

### Fine needle biopsy and FACS analysis

Prior to sample handling, QuantiBRITE® (PE) control beads (340495, BD Biosciences) were used according to the manufacturer’s instructions for later quantification of bound anti-CD20 antibodies. The biopsy sample was resuspended in NH_4_Cl for red blood cell lysis and washed in PBS (5% FBS). Thereafter, filtration through a 35 μm filter (BD Biosciences) was performed. Isotype control stainings were: PE mouse IgG1 (400112-MOPC21, BioLegend), APC mouse IgG1 (400120-MOPC21, BioLegend) and APC/Cy7 mouse IgG1 (400128-MOPC21, BioLegend). The remaining sample was analysed with: FITC anti-kappa or anti-lambda (depending on identified sample clonality on diagnostic biopsy) (F0434/F0435, Dako), APC anti-CD3 (344812-SK7, BioLegend), APC/Cy7 anti-CD19 (557791-SJ25C1, BD Biosciences), PE anti-CD20 QuantiBRITE (347220-L27, BD Biosciences) and DRAQ7 (abcam), added just before samples were sorted utilizing a FACS Aria IIu (BD Biosciences) using a 100 μm nozzle. Cell viability was assessed by FSC/SSC and DRAQ7 showing a negligible amount of dead cells in all samples. Using the Geometric mean of the PE signal the number of bound CD20 antibodies per cell was calculated using QuantiBRITE® (PE) control beads, GraphPad Prism and Microsoft Excel software.

Sorted kappa or lambda positive populations ranged between 30,000 to 1,500,000 cells (median 300,000). Sorted cells were resuspended in RLT+ Lysis buffer (Qiagen) with DTT and stored at -80°C.

### Isolation of PBMCs

PBMCs were isolated from heparinized peripheral blood samples by Lymphoprep according to the manufacturer’s instructions (1114740, Axis-Shield), using RPMI-1640 (Gibco) for sample dilution. Cells were pelleted and stored at -80°C.

### Western blotting

Cells were lysed in Laemmli buffer (Bio-Rad) with β-mercaptoethanol (Scharlau) and protease inhibitor and phosphatase-stop (Roche). Samples were sonicated on a Diagenode Bioruptor system (10 min), heated (99°C, 5 min) and centrifuged (4°C, 5 min) before loaded (equivalent to 100,000 cells per well) onto precast TGX gels (Bio-Rad). Membrane was blocked with 5% dry milk powder solution (w/v) and then incubated with primary and HRP-conjugated secondary antibodies in 1% blocking solution (w/v). Membrane was developed using ECL (Biological Industries). Resulting blots were imaged on a ChemiDoc XRS+ (Bio-Rad) and bands of selected epigenetic biomarkers were normalised via expression of GAPDH. Fold change was determined by normalisation to expression from day 0 samples.

### Quantitative PCR

Total RNA was extracted by AllPrep Micro kit (Qiagen) according to manufacturers protocol. Total RNA was reversely transcribed to cDNA by MultiScript reverse transcriptase from the High Capacity cDNA Reverse Transcription kit (Applied Biosystems). Quantitative real-time PCR analysis was performed in a 20 μL reaction volume containing cDNA, TaqMan Universal PCR Master Mix (Applied Biosystems), and the probes for CD20 (Hs00544819_m1, Life Technologies) and GAPDH (Hs99999905, Life Technologies). The relative quantitative method was used for analysis, and fold change was used to present data.

## Abbreviations

HDACi: Histone deacetylase inhibitor
DLBCL: Diffuse large B-cell lymphoma
(R-) CHO(P): (Rituximab-), cyclophosphamide, anthracyclin doxorubicin, vincristine (and prednisone)
MFI: Mean fluorescence intensity
PBMC: Peripheral blood mononuclear cells
GABA: Gamma-Aminobutyric acid
FNB: Fine needle biopsy
H3K9ac: Histone 3 Lysine 9-acetylation
H3K4me3: Histone 3 Lysine 4-trimethylation.
